# Prognostic value of dual T1 mapping to predict adverse events in TAVR-patients: extra cellular volume as a possible predictor for peri- and post-TAVR adverse events

**DOI:** 10.1186/1532-429X-18-S1-O64

**Published:** 2016-01-27

**Authors:** Jonathan Nadjiri, Eva Hendrich, Albrecht Will, Costanza Pellegrini, Oliver Husser, Christian Hengstenberg, Stefan Martinoff, Martin Hadamitzky

**Affiliations:** 1 grid.6936.a0000000123222966Department of Cardiovascular Diseases, Deutsches Herzzentrum München, Munich, Germany; 2 grid.6936.a0000000123222966Institute of Diagnostic and Interventional Radiology, Klinikum rechts der Isar, Technische Universität München, Munich, Germany; 3 grid.6936.a0000000123222966Department of Radiology and Nuclear Medicine, Deutsches Herzzentrum München, Munich, Germany

## Background

The benefit of a transcatheter aortic valve replacement(TAVR) can differ in patients and therapy bears severe risks. Dual T1 mapping allows for a comprehensive assessment of myocardial tissue by quantification of extracellular volume (ECV) by measuring T1-relaxation before and after administration of Gadolinium(Gd). High degree aortic stenosis can lead to cardiac damage such as diffuse myocardial fibrosis, evaluable by ECV in CMR.

We sought to assess the prognostic value of T1 mapping and ECV to predict adverse events during and after TAVR.

## Methods

Since July 2013, we investigated 105 patients undergoing clinically indicated TAVR by performing additional CMR with T1 mapping sequences. Examinations were performed one day before intervention. We used a Modified Look Locker Inversion Recovery (MOLLI) sequence with 3 inversion pulses and a 4-(1)-3-(1)-2 readout pattern. The post Gd scan for extra cellular volume calculation was performed 10 min. after administration of a bolus of 0.2 mmol/kg body weight gadopentetate dimeglumine. Study endpoints were all cause death, congestive heart failure (CHF) and TAVR associated conduction abnormalities defined as new onset of left bundle branch block (LBBB), AV-Block or implantation of a pacemaker.

## Results

Median follow-up time was 191 days [IQR 59 - 361 days]. Out of 105 patients 97(92%) could be followed. During follow up 10 (10%) patients died, 5 (5%) patients required hospitalization due to CHF, in 17 (18%) patients a pacemaker was required, in 11(11%) patients a new onset of a LBBB was observed and 5(5%) patients had new onset of an AV-Block I. Occurrence of death and CHF had no statistically relevant association with BMI, age, gender, EuroScore or type of valve prosthesis, LV-mass, end diastolic volume, ejection fraction or fluoroscopy time.

ECV was increased (>30%) in 38 patients (40%).

There was no significant correlation between ECV and death, Hazard ratio (HR) 0.847[95% CI: 0.335; 2.14], p = 0.72. ECV in patients with subsequent CHF was higher than in those without an event (33.5 ± 4.6% and 29.1 ± 4.1% respectively), but the difference just did not reach the level of significance HR: 2.16[95% CI: 0.969; 4.84], p = 0.06.

Patients with post-TAVR conduction abnormality (LBBB, AV-block or pacemaker implantation) had statistically relevant lower ECV-values compared to those without an event. Patients with an event had a mean ECV of 28.1 ± 3.16%; patients without an event had a mean ECV of 29.8 ± 4.53, HR: 0.558[95% CI: 0.324; 0.962], p = 0.036.

## Conclusions

In this study, elevated myocardial ECV is a predictor of CHF by trend, CMR may be helpful in identifying patients with a high risk for post-TAVR cardiac decompensation benefitting from an intensified postinterventional surveillance.

Patients with post-TAVR conductions abnormalities have a significantly decreased ECV. One possible explanation of this phenomenon could be that myocardial fibrosis may protect the conduction pathways from pressure caused by the valve prosthesis.

**Figure 1:**
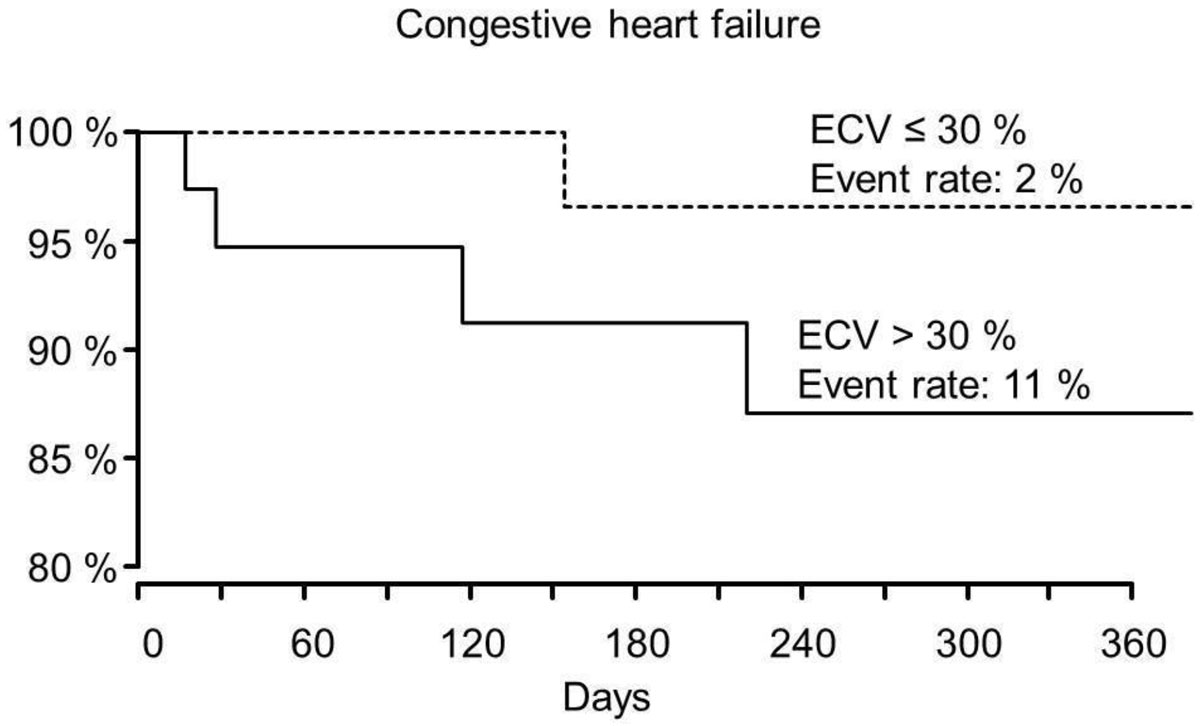
Figure 1

**Figure 2:**
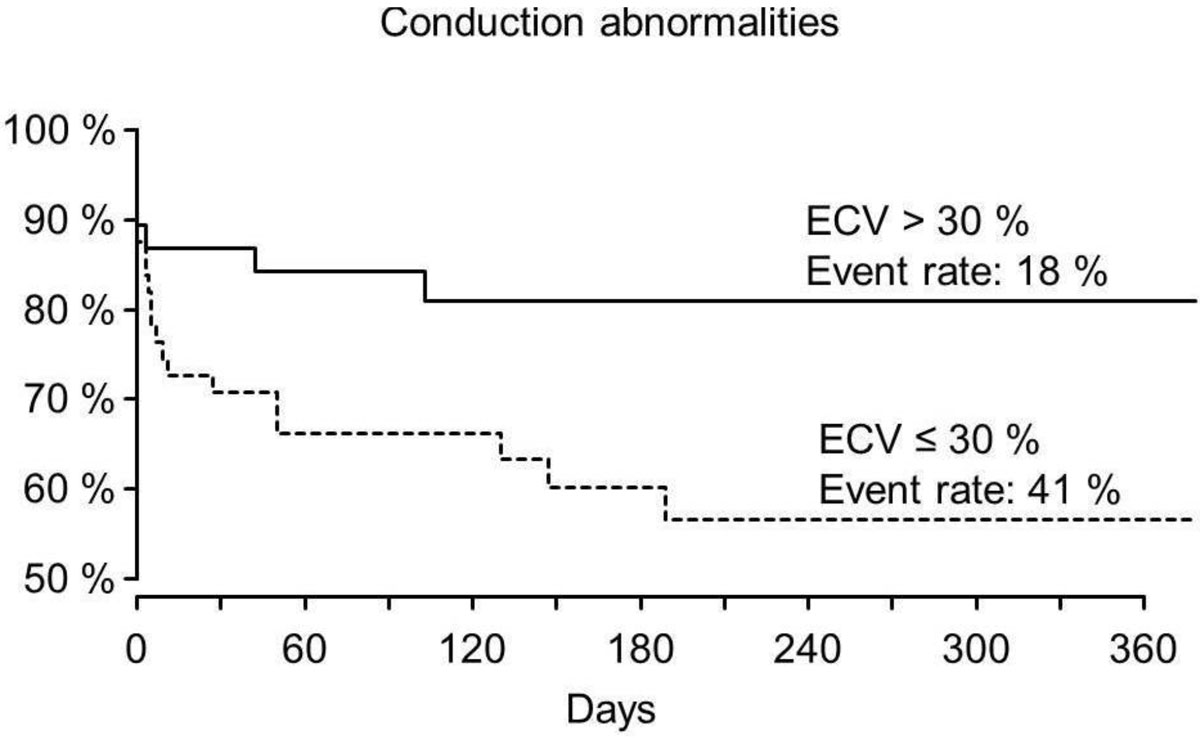
Figure 2

